# Green Removal of DUV-Polarity-Modified PMMA for Wet Transfer of CVD Graphene

**DOI:** 10.3390/nano12224017

**Published:** 2022-11-15

**Authors:** Justinas Jorudas, Daniil Pashnev, Irmantas Kašalynas, Ilja Ignatjev, Gediminas Niaura, Algirdas Selskis, Vladimir Astachov, Natalia Alexeeva

**Affiliations:** 1 THz Photonics Laboratory of Optoelectronics Department, Center for Physical Sciences and Technology (FTMC), Saulėtekis Ave. 3, LT 10257 Vilnius, Lithuania; 2 Department of Organic Chemistry, Center for Physical Sciences and Technology (FTMC), Saulėtekis Ave. 3, LT 10257 Vilnius, Lithuania; 3 Department of Structural Analysis of Materials, Center for Physical Sciences and Technology (FTMC), Saulėtekis Ave. 3, LT 10257 Vilnius, Lithuania; 4 Department of Physical Technologies, Center for Physical Sciences and Technology (FTMC), Saulėtekis Ave. 3, LT 10257 Vilnius, Lithuania

**Keywords:** graphene, DUV, PMMA, THz-TDS

## Abstract

To fabricate graphene-based high-frequency electronic and optoelectronic devices, there is a high demand for scalable low-contaminated graphene with high mobility. Graphene synthesized via chemical vapor deposition (CVD) on copper foil appears promising for this purpose, but residues from the polymethyl methacrylate (PMMA) layer, used for the wet transfer of CVD graphene, drastically affect the electrical properties of graphene. Here, we demonstrate a scalable and green PMMA removal technique that yields high-mobility graphene on the most common technologically relevant silicon (Si) substrate. As the first step, the polarity of the PMMA was modified under deep-UV irradiation at λ = 254 nm, due to the formation of ketones and aldehydes of higher polarity, which simplifies hydrogen bonding in the step of its dissolution. Modification of PMMA polarity was confirmed by UV and FTIR spectrometry and contact angle measurements. Consecutive dissolution of DUV-exposed PMMA in an environmentally friendly, binary, high-polarity mixture of isopropyl alcohol/water (more commonly alcohol/water) resulted in the rapid and complete removal of DUV-exposed polymers without the degradation of graphene properties, as low-energy exposure does not form free radicals, and thus the released graphene remained intact. The high quality of graphene after PMMA removal was confirmed by SEM, AFM, Raman spectrometry, and by contact and non-contact electrical conductivity measurements. The removal of PMMA from graphene was also performed via other common methods for comparison. The charge carrier mobility in graphene films was found to be up to 6900 cm^2^/(V·s), demonstrating a high potential of the proposed PMMA removal method in the scalable fabrication of high-performance electronic devices based on CVD graphene.

## 1. Introduction

Graphene is a unique material with distinctive characteristics that has shown great potential for a wide range of applications, particularly involving its electronic structure and transport properties [1,2,3]. The successful isolation of monolayer graphene by mechanical exfoliation performed in 2004 [4] made it possible to achieve electron mobility, at room temperature, of ~10,000 cm^2^/(V·s). In a free-standing graphene layer, the electron mobility can even reach 200,000 cm^2^/(V·s) at room temperature, the highest value ever known in pure semiconductors [5,6]. Unfortunately, exfoliation typically produces graphene flakes of only tens of micrometer in size, which is not practical for most applications. Different synthesis processes have been intensively studied to provide a route toward the fabrication of graphene of a quality similar to that of exfoliated graphene, but on an industrial scale. The fact that the electronic properties of graphene are altered if it is transferred to a substrate [7], and that the charge carrier mobility drops accordingly by orders of magnitude, makes this task really challenging. Chemical vapor deposition (CVD) is among the most utilized synthesis techniques for the manufacture of single-layer graphene on a large substrate [2,3]; however, the choice of substrate is limited to a very small number of transition metals, such as copper or nickel [8,9,10]. To fabricate graphene-based electronic or optoelectronic devices that require substrates other than these transition metals, additional operations are needed to transfer CVD graphene to the application-compatible substrate [11,12]. This is generally accomplished by dissolving the metal substrate via a wet etching process to release graphene [13,14,15,16,17]. As free-standing graphene is fragile, typically, a supporting layer such as polymethyl methacrylate (PMMA) is used [18,19]. After the transfer process the polymeric transfer agent must be eventually removed via extensive cleaning. However, because PMMA interacts strongly with graphene, complete removal of the PMMA is a difficult task. The residues of PMMA and its products, which are formed during and remain after processing in a solvent, cannot later be removed and will inevitably affect the graphene performance [20,21]. It has been acknowledged by many researchers [22] that the PMMA and its residuals cannot be completely removed by any solvent alone [21], even strong, toxic, and carcinogenic solvents such as anisole [23], acetic acid [24], chlorobenzene [25], and chloroform [26]. Graphene is extremely sensitive to adsorbates and molecules in contact with its surface; hence, the residues tend to act as a dominant source of free charge carriers and scattering centers, suppressing charge transport in graphene [21,27,28,29]. For example, the carrier mobility of as-prepared CVD graphene, 10^3^ to 10^4^ cm^2^/(V·s), decreased to 2 × 10^2^–2.5 × 10^3^ cm^2^/(V·s) after the transfer and PMMA removal in acetone [30]. Therefore, thorough removal of the PMMA residues is crucial for improving the electrical and optoelectronic characteristics of graphene-based devices.

Various strategies have been proposed to ensure effective PMMA removal after graphene transfer and preservation of the unique properties of graphene by maintaining its structural integrity [31,32,33]. One way to overcome incomplete PMMA removal is to reduce the strong interaction of PMMA with graphene by breaking the chain of polymers into lower molecular weight fragments, increasing their solubility as solvent molecules penetrate more easily. Polymer decomposition can be initiated thermally by its depolymerization at 300–400 °C into volatile monomers [34,35] under annealing conditions, including an air, vacuum, or inert atmosphere [36,37,38,39]. However, many reports claim that annealing could not completely remove PMMA residues or other contaminants from the graphene surface [22]. Moreover, defects and doping can be introduced into the graphene lattice during the post-annealing process [39]. Polymer decomposition can also be achieved by oxygen plasma treatment [40], which is a standard method of suppressing PMMA residues in the device processing, with the exception of carbon electronics, since it etches carbon material in the form of graphene, damaging it, introducing strains and distorting the graphene lattice, thus causing both topological and chemical defects. It is possible to break the polymer chain into fragments with a lower molecular weight by irradiating it, which leads to so-called PMMA degradation. Various radiation sources with high energy, such as gamma rays, X-rays, electron beams, proton beams, and ion beams, are used for this purpose [25,41,42,43,44]. However, along with chain scission, another chemical reaction occurs at larger doses, known as crosslinking, which reduces solubility. The resulting net effect on the polymer is determined by the dominance of one reaction over the other [42]. In addition, defects in graphene can be generated if high radiation doses are used. Moreover, the low-electric field electron mobility, achieved using the techniques described earlier, remains mainly at the level of 3 × 10^3^–4 × 10^3^ cm^2^/(V∙s), which until now was the upper limit of the devices fabricated of CVD-grown graphene [45].

In order to ensure the stability of graphene’s chemical composition and structure, and achieve reasonable mobility, scientists have turned attention to the degradation of the polymer under relatively mild processing conditions [39]. As early as the 1990s, researchers have studied the use of deep-ultraviolet (DUV) exposure of PMMA as an alternative to high-energy exposure methods [46,47], demonstrating that mainly the main-chain scissions occur in PMMA during the exposure of polymer to light with wavelengths shorter than 320 nm. Deep-UV exposure with a wavelength of 254 nm is relatively mild compared to other PMMA exposure techniques, which allows one to ensure the integrity of graphene [25,48]. Furthermore, the DUV method excels in terms of simplicity and cost-effectiveness because DUV of 254 nm can be produced using inexpensive low-pressure mercury vapor lamps [25] or UV-C light emitting diodes, which currently are developing rapidly for germicidal applications [49]. A few papers have reported different UV treatment methods already, showing good cleaning [12,22,25,50,51] of DUV-exposed PMMA by acetone, as a liquid [25] or a vapor [52], and by methyl isobutyl ketone (MIBK) mixed or unmixed with IPA [51]. A clean, uniform, and continuous graphene, with typical low sheet resistance and improved contact between graphene and electrodes, was obtained following DUV exposure [25]. However, the aforementioned solvents themselves introduce additional *p*-doping into graphene, changing its electrical properties in an undesirable way.

On the other hand, regarding the quality of graphene detected after the removal of PMMA, an aqueous solution of isopropyl alcohol (IPA) looks promising as a solvent, since by its nature it does not introduce additional residuals that dope graphene, as it can be seen after using other solvents for PMMA removal [53]. The aqueous mixture of IPA, a binary polar solvent, in a certain range of relative concentrations, was introduced in 1988 [54], and it was shown to be an excellent developer for PMMA [55], exposed to an electron beam [55,56,57] or X-ray radiation [44,58], when used as a positive resist in e-beam lithography for the micro- and nanoelectronics. In general, such IPA/water solution has found its wide applications in many areas, as personal care, biomedical, e.g., drug delivery [59] and in principle, mixtures of various alcohols with water can be used [60]. Another important advantage of the aqueous alcohol solvent is non-toxicity. Global production of chemicals has increased fiftyfold since 1950 and is projected to triple again by 2050 compared to 2010 [61]. Humanity is currently operating outside the planetary boundary based on the weight-of-evidence for several of the control variables established by scientists [62]. Under these conditions, reducing the burden of chemicals on the environment in general is becoming critical. Unlike acetone, chloroform, anisole, toluol, MIBK, traditionally used to dissolve PMMA, alcohol-water solvents have much less environmental impact or no effect in the case of ethanol and look very appealing.

In this work, we propose a new approach for the efficient removal of the DUV-exposed PMMA from the surface of wet transferred CVD graphene. Traditionally, the removal of the PMMA film during wet transferring of the CVD graphene implies the dissolution of the polymer (a chain of compounds of the ester type) or the MMA monomer, after thermal or radiational decomposition, with solvents such as acetone (refers to the type of ketones). We propose here to modify this scheme by dissolving the DUV-exposed PMMA with a binary polar solvent of isopropyl alcohol and water. We show that complete dissolution of PMMA is achieved on CVD graphene. As far as we know, the investigation of modification in the polarity of the PMMA under the action of DUV [46,47,48] as well as the mechanism of aqueous isopropyl alcohol advantages as a solvent of the PMMA [59,63] were carried out but independently of each other. The novelty of this work is related to analysis of the mechanism of joint action of these two factors on complete removal of DUV-exposed PMMA from CVD graphene in wet transfer process. Scanning electron microscopy (SEM) and atomic force microscopy (AFM) images, Raman spectra and electrical properties of CVD graphene after PMMA removal were investigated and compared with the samples where PMMA was dissolved using other commonly used solvents such as acetone and chloroform. The proposed new approach is environmentally friendly and does not introduce additional damage to graphene Regarding impact on environment, the proposed approach uses a low-pressure mercury lamp as a source of DUV radiation, while mercury is known to be a toxic metal that affects public health and the environment [64] through bioaccumulation, which can harm humans in various ways [65]. We have used this source for only academic purposes to clearly compare with previous research. We suggest to use UV LEDs as a source that does not contain toxic mercury and have many advantages in terms of energy consumption, lifetime, and compactness [49,66].

## 2. Materials and Methods

To investigate the effects of DUV exposure on PMMA, 996 K molar mass PMMA (Sigma-Aldrich, St. Louis, MO, USA) layers were prepared by spin coating a 4% PMMA solution (0.53 g/10 mL) in anisole (99%, Cole-Parmer, Vernon Hills, IL, USA) to the desired substrate and drying at 180 °C resulting in PMMA film of about 250 nm. For various experimental investigations we have used different substrates. The pristine and DUV-exposed PMMA samples were prepared on a 2 mm-thick fused silica substrates (Eksma Optics, Vilnius, Lithuania) for the investigation of absorbance spectra in UV spectrum range using a UV/VIS spectrometer (Perkin Elmer Lambda 25, Waltham, MA, USA) in the transmission geometry. Changes in IR absorbance in PMMA after DUV exposure were investigated using the FTIR spectrometer (Bruker Vertex v80, Ettlingen, Germany) in the transmission geometry with 1 mm aperture in a vacuum environment with PMMA samples on CaF_2_ substrates of 1 mm thickness (Eksma Optics, Vilnius, Lithuania). To expose PMMA to DUV we have employed commercial crosslinker (Spectronics Corporation XL-1000 UV spectrolinker, Melville, NY, USA) equipped with 254 nm illumination source (UVC Philips TUV T5 lamps), with exposure time of 8 h at the distance between a source and a sample of 16 cm. To characterize the change in the polarity of the exposed PMMA, the optical contact angle meter (KSV Instruments KSV CAM-101, Espoo, Finland) was used to assess the degree of hydrophilicity of the surface.

Finally, sets of graphene samples were prepared for a proof-of-concept of the proposed approach by taking a commercial monolayer CVD graphene synthesized on a copper foil with protective PMMA coating (Graphenea, San Sebastián, Spain) and transferring it on float zone high resistivity (HR) (>10 kΩ∙cm) silicon wafers (Microchemicals, Ulm, Germany). The IPA (≥99.5%, Sigma-Aldrich, St. Louis, MO, USA), acetone (≥99.5%, Chempur, Karlsruhe, Germany) and chloroform (≥99.0%, VWR Chemicals, Radnor, PA, USA) were used as solvents. The quality of the prepared graphene samples was determined by optical microscopy, SEM, atomic force microscopy (AFM), Raman spectroscopy, THz time-domain spectroscopy (TDS), and Hall effect measurements. Scanning electron microscopy images were obtained using secondary electrons at 2 kV and 25 pA beam current in a dual-beam system (FEI Helios Nanolab 650, Hillsboro, OR, USA). The conductive microscopy was performed using scanning probe microscope system (Bruker Dimension 3100, Ettlingen, Germany). Sample bias was set to value of 20 mV using tunneling AFM (TUNA) module under ambient conditions. For these measurements the PtIr cantilever, 300 um length and 0.8 N/m spring constant (type RMN-12PT300B) was used. The samples were characterized by a Raman spectrometer (Renishaw inVia, Wotton-under Edge, UK) using a 1800 lines/mm grating, a microscope equipped with a 50×/0.8 NA objective, and a continuous-wave 532 nm excitation laser operating at a power of 0.6 mW. The beam was focused to the size of a spot with a diameter of 0.8 μm on the sample surface. The position of the Raman spectrum bands on the wavenumber axis was calibrated by a silicon peak at 520.7 cm^−1^. For each sample, 10 spectra were recorded with an integration time of 60 s and used for statistical analysis. The electrical characteristics of graphene were investigated using contactless optical and contact methods. For optical contactless characterization, terahertz time-domain spectroscopy (THz-TDS) was performed with the commercial THz-TDS spectrometer (Teravil T-SPEC 800, Vilnius, Lithuania) in the transmission geometry of a focused beam with a spot size around 2–3 mm. Since THz-TDS measurements only provide information about the conductance of the graphene sheet, contact Hall measurements in a 0.5 T magnetic field (Ecopia HMS-3000, Anyang, Republic of Korea) were also carried out to gain insight into the doping type of the samples.

## 3. Results

### 3.1. Mechanism of Solvation of Exposed PMMA

One of the main points of the proposed method is the change in the properties of PMMA, both chemical and physical, when the polymer is exposed to UV radiation. We show that deep-UV irradiation with a wavelength of 254 nm, used for this purpose, leads not only to photodegradation of the polymer due to the main-chain scissions, as occurs with more powerful irradiation sources, but also the appearance of new species, the formation of which is more pronounced in the case of photodegradation at 254 nm.

**Figure 1:**
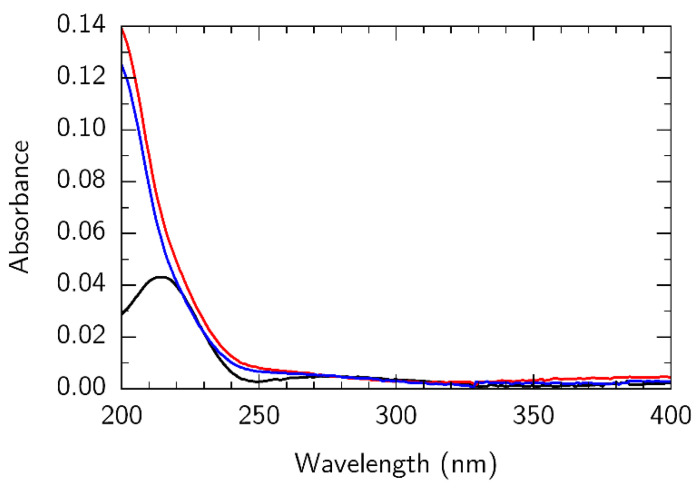
Optical absorption spectra of pristine PMMA/quartz (black line), DUV-exposed PMMA/quartz irradiated in air (red line), DUV-exposed PMMA irradiated in argon (blue line). PMMA film thickness, 0.25 µm.

Since DUV exposure of PMMA takes longer due to the lower energy of irradiation, some products formed after main-chain scissions further absorb UV photons and contribute to the degradation of the polymer by side-chain scissions that are occurring in parallel with the main-chain scissions. As a result, new chemical species are formed, including ketone-type and aldehyde-type carbonyl compounds [47]. To confirm this statement, both pristine and DUV-exposed PMMA was investigated using UV-VIS and FTIR spectroscopy. The changes in UV absorption spectra are shown in Figure 1. Pristine PMMA shows an absorption peak at about 215 nm that, after DUV exposure, becomes more intense and blueshifts out of the range of spectrometer, which makes its exact position hard to evaluate. However, similar behavior was obtained by Shirai et al. [67] using an exposure of 195 nm. Moreover, an absorption band (shoulder) was observed in the pristine PMMA at λ = 275 nm, which is attributed to the carbonyl group in the ester side chain. This feature coincides with that obtained by Torikai et al. [47], where exposure of 254 nm from a low-pressure mercury lamp was used. These results indicate the main-chain scissions in the PMMA sample and the formation of new compounds due to side-chain scissions. To investigate whether our DUV illumination introduces free radicals, which are harmful to graphene, we irradiated PMMA in ambient air and argon environments. The UV-VIS spectra for these samples are shown in Figure 1 as red and blue lines, respectively. The fact that they do not show qualitative differences supports the idea that the observed processes are precisely light-induced, and not chemically induced, for example, by ozone generated by DUV source. Since ozone or free radicals are not formed under such irradiation, the released graphene is expected to remain intact, unlike in higher energy PMMA decomposition methods.

**Figure 2:**
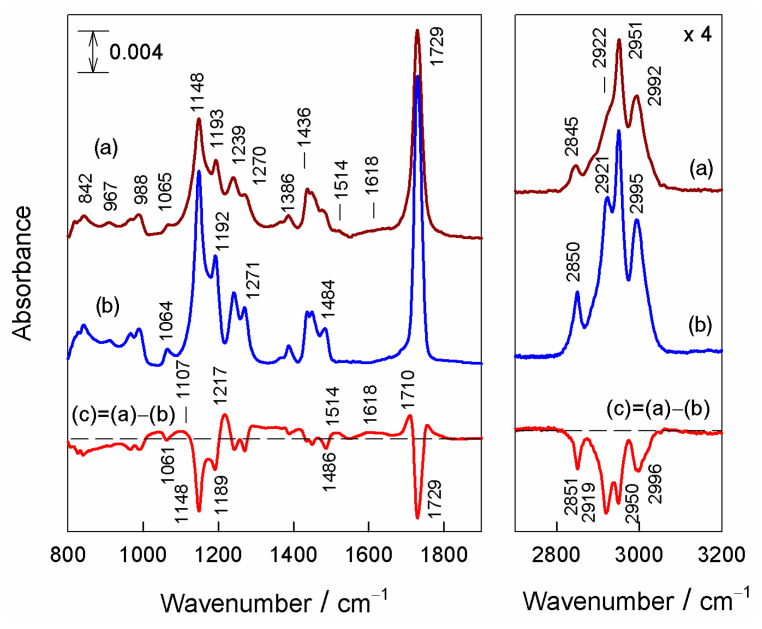
Transmission FTIR spectra of (**a**) DUV-irradiated PMMA/CaF_2_ sample and (**b**) pristine PMMA/CaF_2_ sample. The difference of the two spectra is shown as (**c**). Spectra are shifted vertically for clarity.

To gain insight into the molecular changes of DUV exposure induced in PMMA film, we performed an analysis of FTIR transmission spectra (Figure 2). The dominant absorption bands are associated with the C−H stretching of CH_3_ and CH_2_ groups (2845, 2922, 2951, and 2992 cm^−1^), C=O stretching (1729 cm^−1^), and stretching vibration of the C−O−C group (1148 and 1193 cm^−1^). The vibrational spectrum of PMMA has been analyzed in detail in previous publications [68,69,70,71,72,73,74]. The difference spectrum reveals the appearance of radiation-induced new absorption bands (positive-going features) and decrease in relative intensity of PMMA bands due to cleavage of some chemical bonds (negative-going features). In the fingerprint spectral region (800–1450 cm^–1^), the relative intensity of the 1148 cm^−1^ band associated with the symmetric stretching vibration of the C−O−C group decreases the most, indicating DUV-radiation-induced cleavage of ester bonds. The negative-going feature at 1729 cm^–1^ due to the C=O stretching vibration of the ester group confirms this scenario. In the C−H stretching vibration spectral region, one can find direct evidence of radiation-induced destruction of CH_2_ groups, as two intense negative-going bands at 2851 and 2919 cm^−1^, associated with symmetric and asymmetric stretching vibrations of the methylene group, respectively, appear in the difference spectrum. In addition, the negative-going band, due to CH_2_ scissoring deformation vibration, is visible near 1486 cm^–1^. These spectral changes indicate cleavage in the vicinity of the –CH_2_−moiety (main chain). The positive-going feature near 1710 cm^−1^ reveals the DUV-exposure-induced formation of new species, most likely containing C=O of aldehyde and/or ketone functional groups. The positive-going broad feature near 1618 cm^−1^ is consistent with the formation of sp^2^-hybridized carbon bonds, aromatic carboxyl groups, and hydroxyl groups [74]. In addition, positive-going features near 1107 and 1217 cm^−1^ support the formation of compounds containing C−O bonds or/and hydroxyl groups. Earlier works investigating similar FTIR spectra of DUV-exposed PMMA also concluded that bands of newly produced species were observed in the IR spectrum, and were attributed to ketone-type and aldehyde-type compounds [47].

Chemical changes in the exposed PMMA structure are manifested in the modification of the physical properties of the final products after the photochemical reaction, specifically in polarity. Namely, the increase in polarity is the first basis of the improved solubility mechanism of PMMA exposed to DUV. After exposure, the newly produced ketone and aldehyde type compounds with a polarity higher than that of the original ester functional group can form H-bonds more efficiently, when acting as an acceptor, namely a carbonyl oxygen acceptor, upon subsequent dissolution.

**Figure 3:**
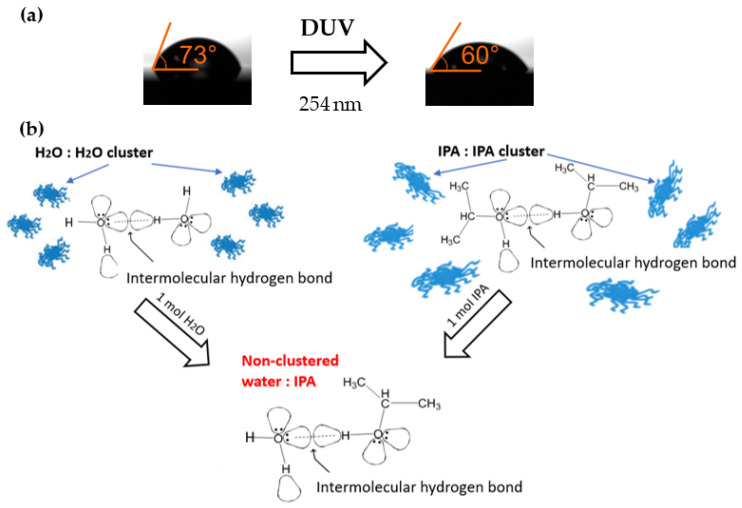
(**a**) Water contact angle on PMMA before (left) and after (right) DUV exposure. Increased hydrophilicity observation corresponds to increasing polarity of new chemical products received after DUV exposure of PMMA. (**b**) Schematic representation of reconstruction of water: water and IPA: IPA clusters into non-clustered water: IPA mixture to act as an effective solvent, where under specific water: IPA mixture due to formation of hydrogen bonds connect effectively to the polymer.

Assessing the polarity of the material is not an easy task, but the water contact angle has been proven to be directly linked to the polarity of the surface [75]. Contact angle measurement is a qualitative way to evaluate whether the surface is hydrophobic or hydrophilic, which is linked to the intermolecular interactions between the surface and a small drop of water. The evaluation of this value gives a clear indication of the modification in polarity of PMMA under the DUV exposure. The contact angles of PMMA were measured before and after exposure to DUV (Figure 3a). The contact angle measurements confirmed that the DUV exposure modified the surface to be more hydrophilic. The decrease in contact angle from the 73.1° on pristine PMMA to 60.2° on exposed PMMA confirms that the initial products of PMMA (ester functional group) are modified into higher polarity products, presumably ketones and aldehydes.

Since higher polarity products form hydrogen bonds, which are much easier than those of pristine PMMA upon subsequent dissolution, it is expected that this will result in the improved removal of PMMA when using a solvent of suitably high polarity. One could expect that the most effective dissolution will be achieved when the solvent with the highest polarity is used, namely, water (relative polarity 0.998) instead of solvents historically used for this purpose such as acetone (C_3_H_6_O 0.355), chloroform (CHCl_3_ 0.259), anisole (C_7_H_8_O 0.198), or toluene (C_7_H_8_ 0.099). However, it is known that PMMA is practically insoluble in water at ambient temperature [59], since water molecules are highly polar, and they form intermolecular connections, hydrogen-bonded clusters, with other water molecules (Figure 3b). Therefore, they have a small physical surface to effectively interact with PMMA (generally, the host polymer), which leads to the insolubility of the polymer [76]. A similar situation occurs between isopropyl alcohol (IPA), or more generally alcohols, and organic compounds with polarity higher than ketones and aldehydes. Alcohols, being organic derivatives of water with one hydrogen atom replaced by an alkyl group, are less polar than water, but can also form intermolecular hydrogen bonds with other IPA molecules, as shown in Figure 3b. Thus, IPA itself is also a poor solvent of PMMA. However, when IPA and water are mixed, the breaking of the intermolecular hydrogen bonds in the water and IPA clusters occurs. The energy required to break the bonds comes from the formation of hydrogen bonds between water and the -OH group of the IPA (hydration of the hydroxyl function of the alcohol/IPA by H-bonding). It is expected that the obtained non-clustered water molecules, as shown in the bottom part of Figure 3b, will have more efficient interactions with PMMA through the “localization” mechanism [76,77], leading to increased polymer solubility. Thus, a binary mixture of polar solvents with an equal number of water and IPA molecules for the formation of a water–alcohol hydrogen bond demonstrates an increased solubility of PMMA. For the IPA/water mixture, the 1:1 molar ratio corresponds to the 7:3 volumetric ratio, the exact ratio at which the maximum solubility of PMMA for IPA/water mixtures has been reported previously [55,63]. Overall, the final products formed after DUV exposure to PMMA (aldehyde and ketone groups) have a higher polarity than the pristine PMMA, and a smaller molecular size, which allows them to form hydrogen bonds with IPA/water mixtures more easily.

### 3.2. DUV-Assisted Wet Transfer of Graphene

**Figure 4:**
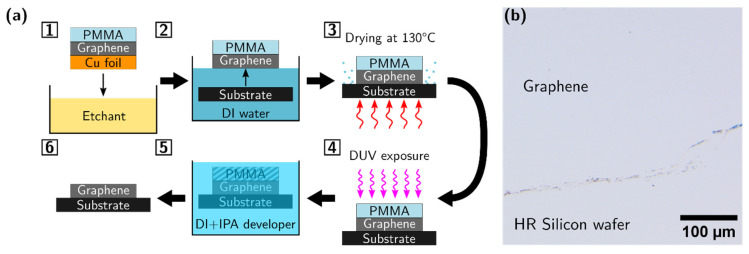
(**a**) Schematic of graphene wet transfer and PMMA removal processing steps. (**b**) Optical microscope image of graphene edge area.

CVD graphene was transferred onto HR Si substrates using a standard wet transfer procedure using PMMA as a support layer [78,79]. The approach is shown schematically in steps 1–3 in Figure 4a, and with the final steps 4 and 5 referred to later in the text as the proposed “DUV method”. First, a Cu foil is etched away in a dilute room temperature RCA standard cleaning 2 (SC-2) solution of 60:5:2 H_2_O/HCl/H_2_O_2_, adapted from [78]. Then, the translucent PMMA/graphene stack is rinsed thoroughly in DI water and wet transferred to a substrate of choice. The prepared PMMA/graphene samples are first dried in ambient conditions for 20 min, then dried in an oven at 130 °C for 30 min. The thickness of the PMMA film was about 60 nm, according to the manufacturer (Graphenea). The PMMA was removed in steps 4 and 5. We used DUV exposure to modify PMMA, and then IPA/water development, expecting no chemical doping of graphene. Namely, after PMMA was exposed to DUV at 254 nm for 3 h (distance between a source and the sample was 16 cm), it was developed in 7:3 volumetric ratio IPA/water mixture for 30 s and dried with N_2_. The result of the entire procedure of transferring and removing of PMMA is shown in Figure 4b, where a clean graphene sample is visible at a purposefully selected graphene edge area on an HR Si wafer.

**Figure 5:**
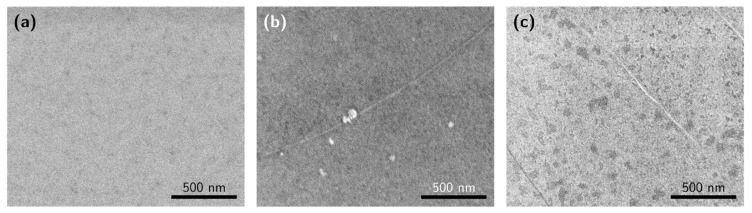
SEM images of the graphene films after different PMMA removal methods: (**a**) DUV exposure and dissolution in IPA/water, (**b**) dissolution in chloroform, (**c**) dissolution in acetone.

To compare the discussed method of PMMA removal, additional PMMA/graphene samples were prepared using the same wet transfer process (steps 1–3), but with PMMA removed with common solvents: acetone and chloroform. A total of five samples were prepared: three samples with DUV treatment and development in aqueous IPA (samples DUV1–DUV3), and one sample each for PMMA removal for 18 h at 23 °C with acetone and chloroform, respectively. As a reference, the standard commercially transferred graphene film to SiO_2_/*p*-Si substrate was also studied. To evaluate the effectiveness of our cleaning procedure, the surfaces of the graphene released with the DUV method and via traditional solvents (chloroform and acetone) were studied via SEM and AFM. SEM images of graphene films prepared by different PMMA removal methods are shown in Figure 5. Comparing the SEM images in Figure 5a–c, graphene processed using chloroform exhibited the highest number of PMMA residuals on the surface. The DUV (Figure 5a) and acetone (Figure 5c) processes showed lower numbers of residuals on the surface.

**Figure 6:**
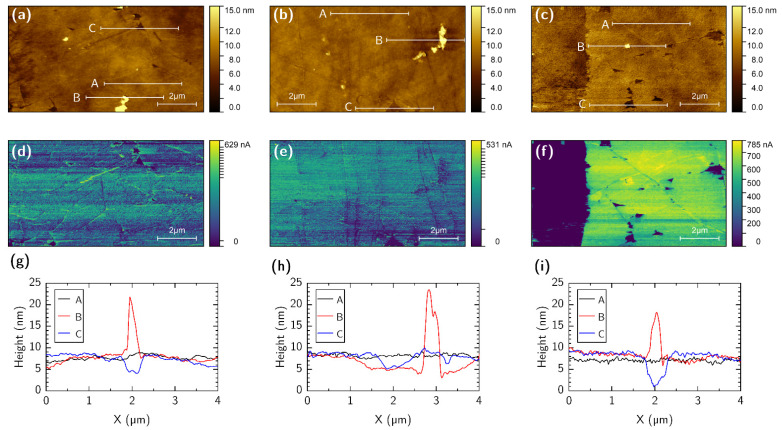
Topography and conductivity images (10 × 5.4 μm^2^) and topography profiles obtained via AFM microscopy of the graphene samples after different PMMA removal methods: (**a**,**d**,**g**) DUV exposure and IPA/water dissolution; (**b**,**e**,**h**) dissolution in chloroform; (**c**,**f**,**i**) dissolution in acetone. Topography profiles (**g**–**i**): A—graphene surface roughness, B—height of contaminants on the graphene, C—hole in graphene showing distance to substrate.

Figure 6 shows topography and simultaneously obtained tunneling current images. Gwyddion software was used to process the AFM images and to extract the surface roughness. The topographical images are shown in Figure 6a for DUV, Figure 6b for chloroform, and Figure 6c for acetone. The DUV cleaning protocol revealed a flat and homogeneous graphene surface with only occasional wrinkles, similar to samples processed with chloroform (18 h soaking). In Figure 6d–f, conductive areas correspond to the graphene film, while non-conductive zones are either the substrate, residuals of the PMMA, or other contaminants. Profiles A (black lines) in Figure 6g–i represents graphene surfaces that were used to calculate root-mean-square roughness values of 0.26 nm for DUV, 0.30 nm for chloroform, and 0.65 nm for acetone. Profile B (red lines) shows the height of the graphene surface contaminants, which were 13.4 nm for DUV, 18.3 nm for chloroform, and 9.8 nm for acetone. The graphene–substrate distances were estimated from profile C (blue lines) to be around 3.9 nm for DUV and chloroform, but for acetone the distance was approximately twofold larger, at 7.9 nm. These results indicate that DUV and chloroform techniques both reveal clean graphene with the roughness of the graphene surface around 0.26–0.30 nm in contrast to standard acetone cleaning.

### 3.3. Raman Spectroscopy of Graphene after PMMA Removal

**Figure 7:**
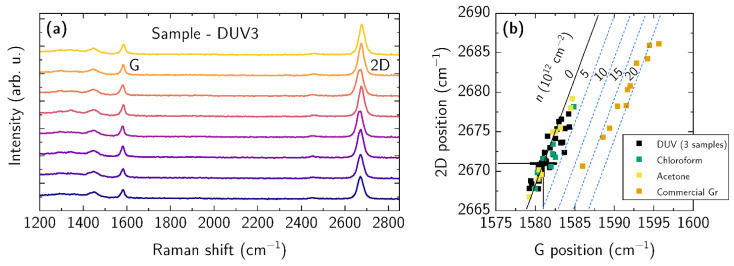
Raman measurements of wet-transferred CVD graphene after PMMA removal: (**a**) Raman spectra of the selected sample DUV3 demonstrating shape and position of G and 2D lines (spectra are shifted vertically for visual clarity). Spectra were measured every 10 µm steps along a line on the graphene. (**b**) Correlation analysis of the G and 2D peak positions for all samples under study, i.e., 3 DUV method samples (black squares), chloroform sample (green squares), acetone sample (yellow squares), and commercially transferred sample (orange squares). The black solid line demonstrates the dependence correlation between the frequencies of the G and 2D lines in the Raman spectrum of undoped graphene under biaxial strain. Blue dashed lines show correlation dependence for doped graphene under biaxial strain. Black cross marks G and 2D line positions in undoped and unstrained graphene.

The Raman spectra for the selected sample DUV3 are shown in Figure 7a. The spectra exhibit two distinct peaks at 1583 cm^−1^ (G peak) and 2674 cm^−1^ (2D peak). It is worth noting that the D peak at around 1350 cm^−1^ falls into elevated background, and was not included in the analysis. To obtain the precise position and FWHM of G and 2D lines, a Lorentz function was used to fit each peak obtaining the mean and standard deviation values of peak intensity and FWHM. The results for all the samples are provided in Table 1. The samples prepared by the DUV method demonstrated a high 2D/G intensity ratio and a low FWHM of the 2D line, found to be up to 2.9 ± 0.22 and below 30 cm^−1^, respectively. The samples prepared using chloroform and acetone exhibited a high 2D/G intensity ratio (>2.4), but their FWHM was larger in comparison with DUV samples, namely, FWHM ≥ 30 cm^−1^. To consider the repeatability of our process in the fabrication of DUV samples, we fabricated three DUV samples over two separate processing runs, the results of which are listed in the Table 1. While the 2D/G ratio and FWHM values of the 2D line fluctuated slightly between the samples, the mean values of FWHM were found in the range of 28 to 30 cm^−1^. Additionally, the fitted positions of the 2D and G lines are used in correlational position analysis, between the 2D and G peaks, which makes it possible to investigate strain and doping in graphene films [80]. The results for the samples obtained in this study and commercially transferred graphene are shown in Figure 7b. The theoretical location (G_0_; 2D_0_) of free-standing undoped graphene is marked with a large black cross, the position of which is at 1581, 2671 cm^−1^. The distribution of positions extends to a line in undoped graphene under biaxial strain, and is plotted as a black line, which is the usual case for graphene transferred onto Si [81]. If a graphene film is *p*-doped and under biaxial strain, this will cause the G peak position to move towards higher Raman shifts and parallel to the strain line. Since the data of the samples prepared in this study (DUV, chloroform, acetone) are distributed between the theoretical lines of undoped graphene and 5 × 10^12^ cm^−2^ doping, this shows a low doping of the prepared graphene samples. A different behavior is observed for a commercially transferred graphene sample. A significant shift in the data points indicates a high doping of graphene films, which was found to be close to 2 × 10^13^ cm^−2^, demonstrating a low sheet resistance of the graphene according to the manufacturer data sheet.

### 3.4. Electrical Properties of Graphene after PMMA Removal

**Figure 8:**
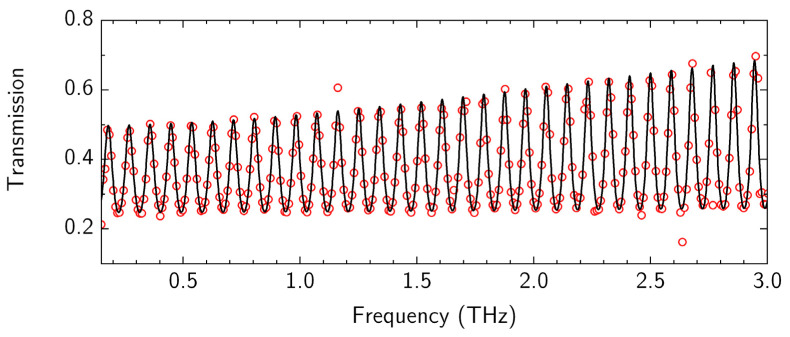
The THz transmission spectrum of graphene on HR Si wafer (DUV3 sample). The experimental data (red circles) was described by Drude conductivity model (black line), the usage of which allows one to estimate the electrical conductivity of the graphene.

Any future applications of CVD graphene require contactless methods of graphene film electrical properties that can conduct large-area mapping and function rapidly [82]. One of such methods is the THz-TDS system, which has been adapted for such applications [83,84,85,86]. The THz transmission spectra were analyzed considering graphene on the dielectric substrate as the delta-thin conductive layer, which is described by the Drude conductivity model, with fitting parameters of sheet conductivity $\sigma_{S}$, scattering time $\tau_{SC}$, and substrate thickness $d_{S}$ [84,85,87]. The transmission spectrum of the graphene sample is shown in Figure 8 by red circles and a fitted curve (black line), which coincide well with experimental points. There are several points (ejections) near the frequencies of 1.1 THz and 2.6 THz, which were caused by natural water vapor variation in ambient air. The transmission spectra of HR silicon were also measured before graphene transfer, demonstrating less dispersion behavior over the frequency range of interest. All measured time-domain characteristics were averaged 500 times.

Relevant electrical properties of the graphene films, such as sheet resistance R_S_, sheet carrier density (unintentional doping) N_S_, and carrier mobility µ, were found from fitting results using the respective definitions:

(1) $$R_{S}=\frac{1}{\sigma_{S}}$$

(2) $$N_{S}=\frac{\pi \hbar^{2}}{qv_{f}{}^{2}}{\left(\frac{\sigma_{S}}{\tau_{SC}}\right)}^{2}$$

(3) $$\mu =\frac{\sigma_{S}}{qN_{S}}$$

The results for all samples are summarized in Table 2. It is important to note that the parameters extracted from THz-TDS results represent statistical averages of any microscale properties over the 2–3 mm spot size of the focused THz beam. All the samples prepared with various PMMA removal techniques exhibited carrier densities below 3.2 × 10^12^ cm^−2^ and mobilities higher than 2410 cm^2^/(V·s). The best overall result was achieved for sample DUV3, which demonstrated very low residual doping and very high mobility values, found to be at the level of 0.8 × 10^12^ cm^−2^ and 6900 cm^2^/(V·s), respectively.

Remote THz characterization is a powerful tool; however, it does not provide information on the type of free-charge carriers that interact with THz radiation. To gain more insight into this, we added Ag epoxy contacts to each corner of the rectangular graphene samples and performed Hall experiments. Two of the three samples prepared using DUV treatment exhibited *p*-type conductivity (DUV1 and DUV2), while the last one demonstrated *n*-type conductivity (DUV3). Samples prepared using chloroform and acetone solvents showed *n* and *p*-type conductivity, respectively. The varying conductivity type for the DUV samples could be a result of slight unintentional doping during PMMA removal, since for all the samples analyzed, carrier density was below 2.5 × 10^12^ cm^−2^. These results indicate that DUV-assisted PMMA removal in an IPA/water solution makes it possible to obtain clean CVD graphene with high charge carrier mobility values, which are required in the field of high-frequency electronics.

## 4. Conclusions

In conclusion, a green and cost-efficient method has been developed for PMMA supportive layer removal from the surface of CVD graphene wet transferred onto an application-compatible substrate of choice, which allows one to obtain scalable graphene with high electron mobility for the needs of high-frequency graphene-based electronics and optoelectronics. In terms of the method, we propose to first modify the polarity properties of PMMA via exposure to deep-ultraviolet (DUV) radiation with a wavelength 254 nm, so that not only the decomposition of PMMA polymer to MMA monomer occurs, as typically happens after thermal or photo-irradiation, but also the formation of final products (ketones and aldehydes) with polarities higher than those of the initial products (esters). Such products form hydrogen bonds much more easily upon subsequent dissolution, which would allow for better removal of PMMA using a solvent of high polarity. We propose to use a binary solvent mixture of isopropyl alcohol and water, which is not only extremely polar, but is also a more environmentally friendly solvent. Generally, an aqueous alcohol—such as ethanol—can be used, especially since it has no environmental impact at all. Another advantage of the method is that low-energy irradiation does not form free radicals, thus the released graphene remains intact, unlike in higher-energy PMMA decomposition methods, such as with an electron beam or even DUV at 195 nm.

The high quality of graphene after the removal of PMMA using our method was confirmed with SEM, AFM, and Raman spectrometry. Electrical transport properties of released graphene, which are of special interest to current research, were fulfilled by means of contactless optical (THz-TDS) and contact (Hall measurements) techniques. It was found that the mobility of charge carriers in graphene films reaches up to 6900 cm^2^/(V·s), which makes the proposed method suitable for removing PMMA from the graphene surface in the industry of high-performance large-scale electronic devices based on graphene.

## 5. Patents

N.A. and I.K. are inventors on a provision patent application filed by the Valstybinis mokslinių tyrimų institutas Fizinių ir technologijos mokslų centras (no. EP3936476A1, published 12 January 2022).

## Figures and Tables

**Table 1:** Properties of graphene Raman spectra prepared by different methods.

Sample	I_2D_/I_G_	FWHM (cm^−1^)	Processing Run
DUV1	2.74 ± 0.31	30.2 ± 1.3	#1
DUV2	3.00 ± 0.41	27.8 ± 1.0	#1
DUV3	2.90 ± 0.22	27.9 ± 1.3	#2
Chloroform	2.45 ± 0.35	29.5 ± 2.2	#2
Acetone	3.13 ± 0.36	31.4 ± 1.2	#2

**Table 2:** Electrical properties of graphene samples prepared by different methods.

Sample	Sheet Resistance R_S_ (Ω/sq.)	Sheet Carrier Density N_S_ (×10^12^ cm^−2^)	Carrier Mobility µ (cm^2^/(V∙s))	Carrier Type	Processing Run
DUV1	670	2.5	3720	*p*	#1
DUV2	840	2.0	3650	*p*	#1
DUV3	1110	0.8	6910	*n*	#2
Chloroform	850	1.3	5890	*n*	#2
Acetone	850	3.1	2410	*p*	#2

## Data Availability

Data available on request.
